# Sistema de saúde do Chile: resposta sanitária e desafios frente à COVID-19

**DOI:** 10.1590/0102-311XPT278525

**Published:** 2026-08-31

**Authors:** Isabel Domingos Martinez dos Santos, Suelen Carlos de Oliveira, Alex Alarcón Hein

**Affiliations:** 1 Instituto de Saúde Coletiva, Universidade Federal Fluminense, Niterói, Brasil.; 2 Escola Nacional de Saúde Pública Sérgio Arouca, Fundação Oswaldo Cruz, Fiocruz, Rio de Janeiro, Brasil.; 3 Escuela de Salud Pública Salvador Allende, Universidad de Chile, Santiago, Chile.

**Keywords:** Sistemas de Saúde, COVID-19, Acesso Universal aos Serviços de Saúde, Reforma dos Serviços de Saúde, América Latina, Health Systems, COVID-19, Universal Access to Health Care Services, Health Care Reform, Latin America, Sistemas de Salud, COVID-19, Acceso Universal a los Servicios de Salud, Reforma de la Atención de Salud, América Latina

## Abstract

O artigo analisa a resposta do sistema de saúde chileno à COVID-19 no período de 2020 a 2023, destacando momentos da trajetória (segundo o contexto epidemiológico e sociopolítico) e as principais medidas. Por fim, traz reflexões acerca das oportunidades e dos limites da resposta à epidemia para a indução de reformas nesse sistema. Realizou-se estudo de caso abrangendo revisão bibliográfica, análise documental e 19 entrevistas semiestruturadas com atores institucionais relevantes. Os resultados indicam que, em meio à instabilidade política, houve investimentos em capacidades sanitárias nacionais. As medidas adotadas evidenciaram a relevância do sistema público de saúde, com gestão centralizada de leitos hospitalares pelo Ministério da Saúde, além de políticas de vigilância em saúde de forma integrada à atenção primária à saúde (APS). A manutenção das capacidades adquiridas e a recuperação da atenção à saúde que fora reprimida foram desafios do momento seguinte, tendo fortalecido na agenda processos de universalização do sistema de saúde via extinção de copagamentos e APS universal.

## Abstract (en)

This study analyzes the Chilean health system response to COVID-19 from 2020 to 2023, highlighting moments (according to their epidemiological and sociopolitical context) and its main measures. It also reflects on the opportunities and limits of the response of this system to the epidemic to reform it. This case study included a literature review, document analysis, and 19 semi-structured interviews with relevant institutional stakeholders. Results indicate that, amidst political instability, national health capacities received investments. The adopted measures highlighted the relevance of the public health system, with centralized management of hospital beds by the Ministry of Health, and integrated health surveillance policies with primary health care (PHC). Maintaining the acquired capacities and recovering the strained health care system constitute challenges after the pandemic, strengthening processes of universalization in the agenda of the health system via universal PHC and by extinguishing co-payments.

## Abstract (es)

Este artículo analiza la respuesta del sistema de salud chileno a la COVID-19 entre el 2020 y el 2023, destacando los momentos clave de su trayectoria (según el contexto epidemiológico y sociopolítico) y las principales medidas adoptadas. Finalmente, ofrece reflexiones acerca de las oportunidades y de los límites de la respuesta a la epidemia para inducir reformas en este sistema. Se realizó un estudio de caso que incluyó una revisión bibliográfica, un análisis documental y 19 entrevistas semiestructuradas con actores institucionales relevantes. Los resultados indican que, en medio de la inestabilidad política, se realizaron inversiones en capacidades sanitarias nacionales. Las medidas adoptadas pusieron de relieve la importancia del sistema público de salud, con una gestión centralizada de las camas hospitalarias por parte del Ministerio de Salud, además de políticas de vigilancia sanitaria integradas con la atención primaria de salud (APS). Mantener las capacidades adquiridas y la recuperación de la atención sanitaria suprimida fueron desafíos en el período siguiente, lo que reforzó la agenda para los procesos de universalización del sistema sanitario mediante la eliminación de copagos y la APS universal.

## Introdução

O Chile sobressai no cenário latino-americano como uma democracia estável, com renda média alta e indicadores socioeconômicos e sanitários elevados ^1^. Em contraste, apresenta elevada concentração de renda, desigualdades socioeconômicas persistentes e tensões político-institucionais ^2^.

Reformas neoliberais na área econômica e social, durante o regime militar (1973-1990), consolidaram um sistema de saúde influenciado pela lógica de mercado e com ampla participação do setor privado ^3^. Caracterizado como dual e segmentado, o sistema de saúde chileno reúne mecanismos públicos e privados no financiamento, asseguramento e prestação de serviços, produzindo trajetórias diferenciadas de acesso conforme renda, vínculo laboral, risco individual e capacidade de pagamento.

Reformas incrementais nos anos 2000, especialmente as Garantias Explícitas de Saúde (GES) baseadas na priorização de uma lista de patologias, objetivaram expandir o acesso e promover a integração; no entanto, não lograram romper com o modelo implantado em décadas anteriores. Permaneceram, como desafios estruturais, a fragmentação institucional e a segmentação social, com repercussões importantes sobre as desigualdades de acesso ^4^.

A vinculação dos cidadãos ao subsistema de saúde público ou ao privado é mediada por cotizações obrigatórias de 7% da renda laboral, podendo ser acrescidas por copagamentos, contratações suplementares e aquisição voluntária de benefícios. O seguro público é gerido pelo Fundo Nacional de Saúde (Fonasa − Fondo Nacional de Salud), cobre cerca de 78,9% da população; o privado, administrado pelas Instituições de Saúde Previdenciária (ISAPRES − Instituciones de Salud Previsional), cobre cerca de 15,3%; e aproximadamente 1,7% está vinculado ao subsistema das Forças Armadas, de Ordem e Segurança Pública (FFAA) ^5^. Nos últimos anos, percebe-se a crescente migração de usuários anteriormente vinculados ao sistema privado para o sistema público, com importante incremento durante a epidemia de COVID-19 ^6^.

Apesar de o sistema público concentrar a maior parte da cobertura populacional, a insuficiência de financiamento público contrasta com os elevados aportes das seguradoras privadas e com os altos gastos diretamente desembolsados ^4^. Em 2019, o gasto total em saúde correspondeu a 9,2% do produto interno bruto (PIB), dos quais 51% corresponderam ao componente público e 48,8% ao privado. Já em 2020, houve elevação para 10,2% do PIB, dos quais 53% corresponderam aos gastos públicos e 47% aos privados, mudanças relacionadas ao enfrentamento da epidemia de COVID-19 ^7^. Entre 2022 e 2024, esse indicador permaneceu estável, sem variações expressivas ^8^.

As capacidades de respostas a emergências sanitárias do sistema de saúde chileno são condicionadas por essa configuração institucional. Destacam-se reformas do sistema de saúde impulsionadas desde 2004, visando ao fortalecimento da autoridade sanitária, formada pelo Ministério da Saúde (Minsal), pelo Instituto de Saúde Pública (ISP) e pelas Secretarias Regionais Ministeriais de Saúde (SEREMI) ^9^. Quanto à atenção primária à saúde (APS), em 2005, buscou-se fortalecê-la como porta de entrada preferencial do sistema, com a introdução do Modelo de Atenção Integral de Saúde, com Enfoque Familiar e Comunitário, criação de modalidades de organização das equipes e incentivos à adscrição territorial da população ^10^.

No Chile, a COVID-19 resultou em 94.028 casos e 2.036 óbitos por milhão de habitantes entre 2020 e 2021. Apesar de indicadores expressivos, a resposta sanitária do período foi destacada por ampla capacidade diagnóstica, expansão da vigilância em saúde e rápida implementação da vacinação. Nesses dois anos, a testagem alcançou a taxa de 1.411 testes por mil habitantes, e a cobertura vacinal já abrangia 86,1% da população ^11^.

A alta capacidade diagnóstica contou com a constituição de rede laboratorial habilitada ao RT-PCR para COVID-19 ^12^. Já a vacinação, iniciada em 2020 para grupos prioritários, obteve reconhecimento internacional ^13^ a partir de 2021, em razão da elevada capacidade de aquisição, do Programa Nacional de Imunização, da cadeia de frio, e uma cultura social pró-vacinação ^14^. Contudo, limites estruturais estavam presentes, especialmente relacionados à segmentação do sistema, à sobrecarga da rede pública e às dificuldades de coordenação da atenção. 

Com a posse de Gabriel Boric (2022-2026), teve lugar uma agenda de processos reformistas do sistema de saúde, orientada pela discussão sobre maior universalização, solidariedade e integração do sistema. Nesse contexto, o artigo tem por objetivo examinar a resposta sanitária chilena à COVID-19 e discutir suas possíveis implicações para os processos de reforma que se seguiram, com base em duas perguntas orientadoras: (i) quais foram os momentos e as principais estratégias da resposta setorial do Chile à COVID-19? (ii) em que medida a resposta sanitária desdobrou-se em oportunidades ou limites para reformas do sistema de saúde?

## Método

Foi realizado um estudo de caso da resposta nacional do sistema de saúde chileno à epidemia de COVID-19, considerando-se o período de 2020 a 2023, correspondente aos primeiros anos da emergência sanitária. A pesquisa baseou-se em referencial de análise de políticas públicas e na literatura sobre resposta de sistemas de saúde à COVID-19 ^15^, principalmente em estudos comparativos em política e sistemas de saúde que enfatizam a análise articulada entre ambiente político institucional, atores, processos decisórios, arranjos organizacionais e resultados das políticas ^16^ ^,^ ^17^. Esse referencial embasou a delimitação dos seguintes eixos de análise: (i) momentos e principais características da resposta setorial à epidemia de COVID-19 - conjuntura, principais atores, marcos e medidas sanitárias no enfrentamento da COVID-19; (ii) movimentos de reformas do sistema de saúde − Plano de Governo, movimento constituinte, “Copagamento Zero” e APS Universal - reflexões com base na resposta sanitária à epidemia. 

A pesquisa foi desenvolvida em duas etapas. A primeira abrangeu análise bibliográfica, conteúdos de mídia e documentos oficiais, especialmente do Ministério da Saúde, outros órgãos nacionais e comissões (listados no Quadro 1).

**Quadro 1:** Relação dos principais documentos oficiais analisados. Chile, 2020-2023.

AUTOR	DOCUMENTO	DATA DE PUBLICAÇÃO	LINK DE ACESSO
Ministério da Saúde	Comunicado na conta oficial do Ministério da Saúde do Chile no Twitter do Ministro da Saúde, Jaime Mañalich, e da Chefe de Epidemiologia, Johanna Acevedo, sobre o Novo Coronavírus e as medidas adotadas no país	20/Janeiro/2020	https://www.pscp.tv/ministeriosalud/1yNGapblqagKj
Ministério da Saúde/Subsecretaria de Saúde Pública	*Decreto nº 4. Declara Alerta Sanitário E Concede Poderes Excepcionais Devido à Emergência Internacional Causada Pelo Novo Coronavírus*	8/Fevereiro/2020	https://www.bcn.cl/leychile/navegar?idNorma=1142163
Ministério da Saúde/Gabinete do Ministro	*Resolução nº 131 Exenta. Cria Grupo de Trabalho Denominado “Consejo Asesor Covid 19”*	11/Março/2020	https://www.camara.cl/estado_excepcion/Anexo%201/03.%20Res%20N%C2%BA%20131%20-%2011.3.pdf
Ministério da Saúde	Minuta da reunião de 15 de março de 2020 - Comité Asesor Covid-19	15/Março/2020	https://pt.scribd.com/document/451813048/Minuta-Comite-Asesor-COVID-15-Marzo-2020#
Ministério do Interior e Segurança Pública/Subsecretaria do Interior	*Decreto nº 104. Declara Estado de Exceção Constitucional de Catástrofe, Por Calamidade Pública, no Território do Chile*	18/Março/2020	https://www.bcn.cl/leychile/navegar?idNorma=1143580
Ministério do Interior	Notícia − *Ministros Blumel, Mañalich y Couve Encabezan Primera Reunión de Mesa Social Covid-19 en La Moneda*	22/Março/2020	https://www.gob.cl/noticias/ministros-blumel-manalich-y-couve-encabezan-primera-reunion-de-mesa-social-covid-19-en-la-moneda/
Colégio Médico do Chile	Minuta da reunião de 24 de março de 2020 - Colégio Médico do Chile − Mesa Social COVID-19	24/Março/2020	https://www.colegiomedico.cl/wp-content/uploads/2020/03/MINUTA-MONEDA-24-MARZO.pdf
Ministério da Saúde/ Subsecretaria de Redes Assistenciais	*Resolução nº 156 Exenta. Dispõe Instruções Para a Coordenação da Rede Pública e Privada de Saúde por Parte da Subsecretaria de Redes Assistenciais*	1/Abril/2020	https://www.bcn.cl/leychile/navegar?idNorma=1143997
Ministério da Saúde/Departamento de Comunicações	Notícia − *Ministro de Salud Informa Sobre Casos Recuperados y Estrategia de Cuarentena Dinâmica*	13/Abril/2020	https://www.minsal.cl/ministro-de-salud-informa-sobre-casos-recuperados-y-estrategia-de-cuarentena-dinamica/
Universidade do Chile/Diário UChile	Notícia - *Alcaldes v/s Gobierno: Más de 50 Jefes Comunales Exigen Fin al “Secretismo” del Minsal*	16/Abril/2020	https://radio.uchile.cl/2020/04/16/alcaldes-vs-gobierno-mas-de-50-jefes-comunales-exigen-mas-informacion-sobre-COVID-19/
Presidência da República	Comunicado - *Presidente Piñera anuncia “Plan Retorno Seguro Hacia una Nueva Normalidad”*	24/Abril/2020	https://prensa.presidencia.cl/lfi-content/uploads/2020/04/24-04-2020-pdte-pinera-anuncia-plan-retorno-seguro-hacia-una-nueva-normalidad-texto-oficial.pdf
Ministério da Ciência, Tecnologia, Conhecimento e Inovação	Informe #1 - Mesa Social, Submesa de Datos COVID-19	6/Maio/2020	https://www.minciencia.gob.cl/legacy-files/informe_1_submesa_datos_covid19_200507.pdf
Cientistas, especialistas e divulgadores de diferentes áreas	Carta aberta ao Presidente da República do Chile: propostas para evitar uma catástrofe por COVID-19	30/Maio/2020	https://drive.google.com/file/d/1mmAFT09hG1tkvXmqmx_S4uDtYXZ5rqWU/view
Universidade do Chile	Anais da Universidade do Chile - Mesa de Conversación “Estado, pandemia y transparência”	9/Julho/2020	https://anales.uchile.cl/index.php/ANUC/article/view/58940/62454
Ministério da Saúde	*Plan Paso a Paso, Nos Cuidamos*	19/Julho/2020	https://www.minsal.cl/wp-content/uploads/2020/07/ConocePlanPasoaPaso.pdf
Universidade do Chile/Diário UChile	Notícia − *Comisión Chilena de DD.HH. por Plan Paso a Paso: “Constituye una Inaceptable Política de Ensayo y Error”*	28/Julho/2020	https://radio.uchile.cl/2020/07/28/comision-chilena-de-dd-hh-por-plan-paso-a-paso-constituye-una-inaceptable-politica-de-ensayo-y-error/
Ministério da Ciência, Tecnologia, Conhecimento e Inovação	*Decreto nº 12. Cria Comissão Assessora Ministerial Científica Para a Disponibilidade de uma Vacina Contra a COVID-19*	30/Julho/2020	https://www.bcn.cl/leychile/navegar?idNorma=1147875
Ministério da Ciência, Tecnologia, Conhecimento e Inovação	Notícia − *Primer ensayo clínico nacional de una vacuna contra el Covid-19 ya cuenta con financiamiento y fecha de inicio*	2/Agosto/2020	https://www.minciencia.gob.cl/noticias/primer-ensayo-clinico-nacional-de-una-vacuna-contra-el-covid-19-ya-cuenta-con-financiamiento-y-fecha-de-inicio/
Ministério da Saúde/Departamento de Comunicações	Notícia − *Presidente Piñera Anuncia Reapertura de Frontera en Aeropuerto Arturo Merino Benítez: “El Coronavirus Sigue Estando Entre Nosotros y, en Consecuencia, No Podemos Descuidarnos”*	22/Novembro/2020	https://www.minsal.cl/presidente-pinera-anuncia-reapertura-de-frontera-en-aeropuerto-arturo-merino-benitez-el-coronavirus-sigue-estando-entre-nosotros-y-en-consecuencia-no-podemos-descuidarnos/
Ministério da Saúde/ Subsecretaria de Saúde Pública	*Protocolo de Coordinación Para Acciones de Vigilancia Epidemiológica Durante la Pandemia de COVID-19 en Chile: Estrategia Nacional de Testeo, Trazabilidad y Aislamiento*	3/Dezembro/2020	https://www.paho.org/sites/default/files/2020-12/MINSAL%2C%202020%2C%20Protocolo%20Estrategia-Testeo-Trazabilidad-y-Aislamiento.pdf
Ministério da Saúde/Subsecretaria de Saúde Pública	*Guía de Autocuidado para Veraneantes*	4/Dezembro/2020	https://www.minsal.cl/wp-content/uploads/2020/12/20201209_Gu%C3%ADa-de-Autocuidado-para-Veraneantes.pdf
Ministério da Saúde/Subsecretaria de Saúde Pública	*Estrategia TTA en Verano*	s.d.	https://www.minsal.cl/wp-content/uploads/2021/01/Documento-Estretegia-en-Verano-TTA.pdf
Ministério da Saúde/Departamento de Comunicações	Notícia − *Presidente Piñera Recibe Primer Cargamento de Vacunas Contra el Covid-19: “Son Una Luz de Esperanza”*	24/Dezembro/2020	https://www.minsal.cl/presidente-pinera-recibe-primer-cargamento-de-vacunas-contra-el-covid-19-son-una-luz-de-esperanza/
Ministério da Saúde/Departamento de Comunicações	Notícia - *Chilenos y Extranjeros Residentes Deben Contar con PCR Negativo para Ingresar al País*	4/Janeiro/2021	https://www.minsal.cl/chilenos-y-extranjeros-residentes-deben-contar-con-pcr-negativo-para-ingresar-al-pais/
Ministério da Saúde/Departamento de Comunicações	Notícia - *Ministro Enrique Paris Destacó Anuncios Presidenciales Respecto al Plan Nacional de Vacunación*	23/Janeiro/2021	https://www.minsal.cl/ministro-enrique-paris-destaco-anuncios-presidenciales-respecto-al-plan-nacional-de-vacunacion/#:~:text=Enrique%20Paris%2C%20valor%C3%B3%20los%20dichos,de%20dosis%20Pfizer%20%E2%80%93%20BioNTech%20aseguradas
Ministério da Saúde/Departamento de Comunicações	Notícia - *Presidente Piñera da a Conocer Calendario de Vacunación Masiva Contra el COVID-19*	28/Janeiro/2021	https://www.minsal.cl/presidente-pinera-da-a-conocer-calendario-de-vacunacion-masiva-contra-el-COVID-19/#:~:text=enero%20de%202021-,Presidente%20Pi%C3%B1era%20da%20a%20conocer%20calendario%20de%20vacunaci%C3%B3n%20masiva%20contra,febrero%2C%20comenzando%20por%20adultos%20mayores
Ministério da Saúde/Departamento de Comunicações	Notícia − *Gobierno se Reunió con Alcaldes de Todo Chile para Coordinar Distribución de Vacunas y Proceso* de *Inoculación Contra el COVID-19*	30/Janeiro/2021	https://www.minsal.cl/gobierno-se-reunieron-con-alcaldes-para-coordinar-distribucion-de-vacunas-y-proceso-de-inoculacion/
Ministério da Saúde/ Subsecretaria de Saúde Pública	*Planificación: Vacunación Contra el SARS-CoV-2*	2/Fevereiro/2021	https://www.minsal.cl/wp-content/uploads/2021/02/Planificaci%C3%B3n-vacunaci%C3%B3n-contra-SARS-CoV-2-02-02-2021.pdf
BBC News Mundo	Notícia − *Coronavirus en Chile: el País Cierra sus Fronteras Ante el Aumento de los Casos de COVID-19*	1/Abril/2021	https://www.bbc.com/mundo/noticias-america-latina-56611784
ICOVID Chile	Informe − *ICOVID en Alerta: Sistema Hospitalario no Resiste un Fin de Semana Largo con Alta Movilidad*	1/Abril/2021	https://uploads.strikinglycdn.com/files/c6dc7528-ae09-4cb4-a256-7c5437342df0/Informe%2034_ICOVID_Chile.pdf?id=3396045
Ministério da Saúde/Conselho Assessor COVID-19	Minuta 21 de maio de 2021 - *Sobre Reducir Restricciones a Personas Con Esquema de Vacunación Completo para Actividades de Bajo Riesgo*	21/Maio/2021	https://drive.google.com/file/d/1GNcxLr6BA1NnLt6StMqrIDxpOaKhQ5qH/view
Universidade do Chile/Diario UChile	Notícia − *Presidente Piñera Anuncia Implementación de “Pase de Movilidad” para Vacunados*	23/Maio/2021	https://radio.uchile.cl/2021/05/23/presidente-pinera-anuncia-implementacion-de-pase-de-movilidad-para-vacunados/
Senado da República do Chile	Notícia − *Hasta el 30 de Septiembre Regirá Nuevo Estado de Excepción Constitucional*	24/Junho/2021	https://www.senado.cl/comunicaciones/noticias/hasta-el-30-de-septiembre-regira-nuevo-estado-de-excepcion-constitucional
La República	Notícia − *Chile Extiende Cierre de Fronteras Para Viajeros Ante la Entrada de la Variante Delta*	25/Junho/2021	https://www.larepublica.co/globoeconomia/chile-extiende-cierre-de-fronteras-para-viajeros-ante-la-entrada-de-la-variante-delta-3191579
Ministério da Saúde	*Plan Paso a Paso, Nos Cuidamos* (atualização)	8/Julho/2021	https://observatorio.medicina.uc.cl/wp-content/uploads/2021/07/Actualizaci%C3%B3n-PASO-A-PASO.pdf
Ministério da Saúde/Subsecretaria de Saúde Pública	*Resolução nº 672 Exenta − Estabelece o Plano “Fronteras Protegidas”*	24/Julho/2021	https://www.bcn.cl/leychile/navegar?idNorma=1162929
Transporte Informa	Notícia *− Implementan el Plan de «Fronteras Protegidas»*	28/Julho/2021	https://www.transporteinforma.cl/implementa-el-plan-de-fronteras-protegidas/
Ministério da Saúde/Comité Asesor COVID-19	Minuta da reunião de 2 de agosto de 2021 - Comité Asesor COVID-19	2/Agosto/2021	https://drive.google.com/file/d/1eeK0HcXYor7K4r6D8UD9tUYwbnIUUF7v/view
Ministério da Saúde/Departamento de Comunicações	Notícia − *Vacunas Contra SARS- CoV-2 Utilizadas en Chile Mantienen Altos Niveles de Efectividad Para Evitar Hospitalización, Ingreso a UCI y Muerte*	3/Agosto/2021	https://www.minsal.cl/vacunas-contra-sars-cov-2-utilizadas-en-chile-mantienen-altos-niveles-de-efectividad-para-evitar-hospitalizacion-ingreso-a-uci-y-muerte/
Ministério da Saúde/Departamento de Comunicações	Notícia − *Ministro de Salud Valora Anuncio de Laboratorio Chino Sinovac de Instalar Una Planta de Vacunas en Chile*	4/Agosto/2021	https://www.minsal.cl/ministro-de-salud-valora-anuncio-de-laboratorio-chino-sinovac-de-instalar-una-planta-de-vacunas-en-chile/
Ministério da Saúde/Departamento de Comunicações	Notícia − *Presidente Piñera da Inicio a la Vacunación Con Dosis de Refuerzo Contra COVID-19*	11/Agosto/2021	https://www.minsal.cl/presidente-pinera-da-inicio-a-la-vacunacion-con-dosis-de-refuerzo-contra-el-COVID-19/
Ministério da Saúde/Departamento de Comunicações	Notícia − *COVID-19: Gobierno Anuncia Fin del Estado de Excepción*	27/Setembro/2021	https://www.minsal.cl/COVID-19-gobierno-anuncia-fin-del-estado-de-excepcion/#:~:text=El%20Presidente%20Sebasti%C3%A1n%20Pi%C3%B1era%20anunci%C3%B3,18%20de%20marzo%20de%202020
Ministério da Saúde/Departamento de Comunicações	Noticia − *COVID-19: Región Metropolitana Retrocede a Preparación*	25/Outubro/2021	https://www.minsal.cl/COVID-19-region-metropolitana-retrocede-a-preparacion/
Subsecretaria de Turismo	Notícia − *Plan Fronteras Protegidas: Extranjeros Con Dosis de Refuerzo Validada Ante el Minsal se Eximirán de PCR al Ingreso a Chile*	15/Novembro/2021	https://www.subturismo.gob.cl/2021/11/15/plan-fronteras-protegidas-extranjeros-con-dosis-de-refuerzo-validada-ante-el-minsal-se-eximiran-de-pcr-al-ingreso-a-chile/
Ministério da Saúde/Departamento de Comunicações	Notícia − *COVID-19: Gobierno Anuncia Cambios al Plan Fronteras Protegidas*	29/Novembro/2021	https://www.minsal.cl/COVID-19-gobierno-anuncia-cambios-al-plan-fronteras-protegidas/
Ministério da Saúde/Departamento de Comunicações	Noticia − *COVID-19: Ministerio de Salud Informa Sobre Protocolo Ante Variante Ómicron*	9/Dezembro/2021	https://www.minsal.cl/COVID-19-ministerio-de-salud-informa-sobre-protocolo-ante-variante-omicron/
Ministério da Saúde/Departamento de Comunicações	Notícia − *COVID-19: Gobierno Posterga Apertura de 5 Pasos Fronterizos*	30/Dezembro/2021	https://www.minsal.cl/COVID-19-gobierno-posterga-apertura-de-5-pasos-fronterizos/
Coalizão “Apruebo Dignidad”	*Programa de Gobierno − Apruebo Dignidad*/Boric Presidente	2021	https://observatorioplanificacion.cepal.org/sites/default/files/plan/files/Plan%2Bde%2Bgobierno%2BAD%2B2022-2026%2B%282%29.pdf
Ministério da Saúde/Instituto de Saúde Pública (ISP)	*ISP: Memoria de los dos Primeros Años de la Pandemia*	março/2022	https://www.ispch.cl/wp-content/uploads/2022/04/MemoriaPandemia.pdf
Ministério da Saúde/ Departamento de Comunicação e Relações Públicas	*COVID-19 en Chile: Pandemia 2020-2022*	7/Março/2022	https://www.minsal.cl/wp-content/uploads/2022/03/2022.03.03_LIBRO-COVID-19-EN-CHILE-1-1.pdf
Governo do Chile	Notícia − *Ministerio de Salud Ajusta el Criterio Para Contabilizar Personas Fallecidas por COVID-19*	21/Março/2022	https://www.gob.cl/noticias/ministerio-de-salud-ajusta-el-criterio-para-contabilizar-personas-fallecidas-por-covid-19/
Governo do Chile	Notícia − *COVID-19: Subsecretario de Salud Pública Informa Sobre Una 3Mejoría en el Esce.nario Epidemiológico en el País*	29/Março/2022	https://www.gob.cl/noticias/covid-19-subsecretario-de-salud-publica-informa-sobre-una-mejoria-en-el-escenario-epidemiologico-en-el-pais/
Ministério da Saúde/ Subsecretaria de Saúde Pública	*Resolução nº 473 exenta. Cria a Comissão Nacional de Resposta Pandêmica COVID-19 e Extingue o Conselho Assessor COVID-19*	2/Abril/2022	https://www.bcn.cl/leychile/navegar?idNorma=1174349&idVersion=2022-09-29
Ministério da Saúde/ Subsecretaria de Saúde Pública	*Cuarta Dosis de Vacuna Contra Sars-Cov-2 (Población General Desde los 18 años)* (atualização)	6/Junho/2022	https://www.minsal.cl/wp-content/uploads/2022/06/Cuarta-dosis-de-vacuna-contra-SARS-CoV-2-en-poblacio%CC%81n-general-desde-los-18-an%CC%83os-ACTUALIZACIO%CC%81N.pdf
Governo do Chile	Notícia − *Ministerio de Salud Presentó el Consejo para la Universalización de la Atención Primaria de Salud*	14/Julho/2022	https://www.gob.cl/noticias/ministerio-de-salud-presento-el-consejo-para-la-universalizacion-de-la-atencion-primaria-de-salud/?utm_source=chatgpt.com
Ministério da Saúde/Subsecretaria de Saúde Pública	*Plan Seguimos Cuidándonos, Paso a Paso* (atualização)	25/Agosto/2022	https://www.minsal.cl/wp-content/uploads/2022/04/2022.08.25_PLAN-SEGUIMOS-CUIDANDONOS-PASO-A-PASO.pdf
Ministério da Saúde/Subsecretaria de Saúde Pública	*Resolução nº 1.112 exenta. Altera a Resolução Exenta nº 176/1999 do Ministério da Saúde, que Aprova a Tabela de Valores (Arancel) das Prestações de Saúde do Livro II do DFL nº 01/2005 do Ministério da Saúde*	29/Agosto/2022	https://www.bcn.cl/leychile/navegar?idNorma=1180513
Governo do Chile	Notícia − *Chile Avanza a Fase de “Apertura” en el Manejo de la Pandemia COVID-19*	21/Setembro/2022	https://www.gob.cl/noticias/chile-avanza-a-fase-de-apertura-en-el-manejo-de-la-pandemia-covid-19/
Ministério da Saúde/Subsecretaria de Saúde Pública	*Resolução nº 1.400 Exenta. Estabelece o Plano “Seguimos Cuidándonos Paso a Paso”*	29/Setembro/2022	https://www.minsal.cl/wp-content/uploads/2022/04/Paso-a-paso-publicado.pdf
Ministério da Saúde/Subsecretaria de Saúde Pública	*Decreto nº 75 − Prorroga a Vigência e Altera o Decreto nº 4/2020 do Ministério da Saúde, que declara alerta sanitária por um período definido e concede poderes extraordinários devido à emergência de saúde pública internacional causada pelo surto do* novo Coronavírus *(2019-nCoV)*	30/Setembro/2022	https://www.bcn.cl/leychile/navegar?idNorma=1182131
Convención Constitucional	*Propuesta Constitución Política de la República de Chile*	2022	https://www.chileconvencion.cl/wp-content/uploads/2022/07/Texto-Definitivo-CPR-2022-Tapas.pdf (acesso restrito)
Superintendencia de Seguridad Social	*Licencias médicas aumentan en un 14,7% y alcanzan las 9,3 millones en 2022*	27/Janeiro/2023	https://www.suseso.cl/605/w3-article-701967.html
Ministério da Saúde/Subsecretaria de Saúde Pública	*Decreto nº 10. Prorroga a Vigência do Decreto nº 4/2020 do Ministério da Saúde, que Declara Alerta Sanitária Por Período Determinado e Concede Poderes Extraordinários Diante da Emergência de Saúde Pública Internacional Causada Pelo Novo Coronavírus (2019-nCoV)*	23/Março/2023	https://www.bcn.cl/leychile/navegar?idNorma=1190518
Governo do Chile	Notícia − *Balance Covid-19: Casos Nuevos Disminuyen en Más de un 40%*	12/Abril/2023	https://www.gob.cl/noticias/cifras-covid-baja-cantidad-casos-nuevos-reporte-minsal/
Conselho Constitucional	*Propuesta Constitución Política de la República de Chile*	2023	https://www.procesoconstitucional.cl/docs/Propuesta-Nueva-Constitucion.pdf

TTA: testagem rastreamento e isolamento.

Fonte: elaboração própria.

A segunda etapa compreendeu entrevistas semiestruturadas com atores envolvidos na resposta à COVID-19 e nos processos de reforma em curso. Foram realizadas 19 entrevistas, em dois momentos: entre 06 de janeiro e 11 de fevereiro de 2023 e 09 e 13 de dezembro de 2024, tendo como eixos centrais a condução político-institucional da resposta sanitária, a atuação dos principais atores, a articulação público-privada na ampliação da capacidade assistencial e suas repercussões sobre a agenda de reformas do sistema de saúde chileno. Ressalte-se que duas pessoas foram entrevistadas ao mesmo tempo em duas das 19 entrevistas. O perfil dos entrevistados consta do Quadro 2, evitando-se a sua identificação direta.

**Quadro 2:** Perfil dos entrevistados segundo inserção institucional.

Entrevista	Instituição/Departamento ou Setor
E1	Escuela de Salud Pública/Universidad de Chile
E2	Escuela de Salud Pública/Universidad de Chile
E3	Entidade Profissional − Colegio Médico de Santiago
E4	OPAS/OMS Chile
E5	Instituto de Salud Pública
E6	Minsal − Departamento de Epidemiología
E7	Minsal − Departamento de Epidemiología Oficina Reglamento Sanitario Internacional
E8	Minsal − Departamento de Epidemiología
E9	Minsal − División de Planificación Sanitaria
E10	Minsal − Departamento de Epidemiología
E11	Minsal − SEREMI de salud
E12	Minsal − SEREMI de salud
E13	Minsal − SEREMI de salud
E14	Minsal − Servicio de Salud − APS
E15	Minsal − APS
E16	Minsal − APS
E17	CESFAM
E18	Fonasa
E19	Superintendencia de Salud

APS: atenção primária à saúde; CESFAM: Centros de Saúde Familiar; Fonasa: Fundo Nacional de Saúde; Minsal: Ministério da Saúde Chileno; OMS: Organização Mundial da Saúde; OPAS: Organização Pan-Americana da Saúde; SEREMI: Secretarias Regionais Ministeriais de Saúde.

Fonte: elaboração própria (2024).

O estudo foi submetido e aprovado pelo Comitê de Ética em Pesquisa da Escola Nacional de Saúde Pública Sergio Arouca da Fundação Oswaldo Cruz (ENSP/Fiocruz, sob o parecer nº 54755321.0.0000.5240) em conformidade com as diretrizes da *Resolução nº 466/2012* do Conselho Nacional de Saúde.

## Resultados 

### Primeiros anos da resposta sanitária à epidemia de COVID-19 no Chile: tensões políticas, centralidade nacional e investimentos em capacidades institucionais 

Os primeiros anos da resposta sanitária à COVID-19 no Chile (2020-2021) ocorreram durante o segundo mandato de Sebastián Piñera (2018-2022), em contexto de forte instabilidade político-institucional, marcado por protestos sociais (*estallido social*) iniciados em 2019 e pelo subsequente processo constituinte que perdurou após o fim do mandato presidencial. A crise expressou críticas históricas: às desigualdades estruturais; à segregação nos sistemas de saúde e educação; ao sistema previdenciário privado; e à crise de representação política. Inicialmente, o governo respondeu com agenda de reformas e uso da força, culminando em um acordo político, em novembro de 2019, criticado como um pacto da “classe política”. 

**Figura 1:**
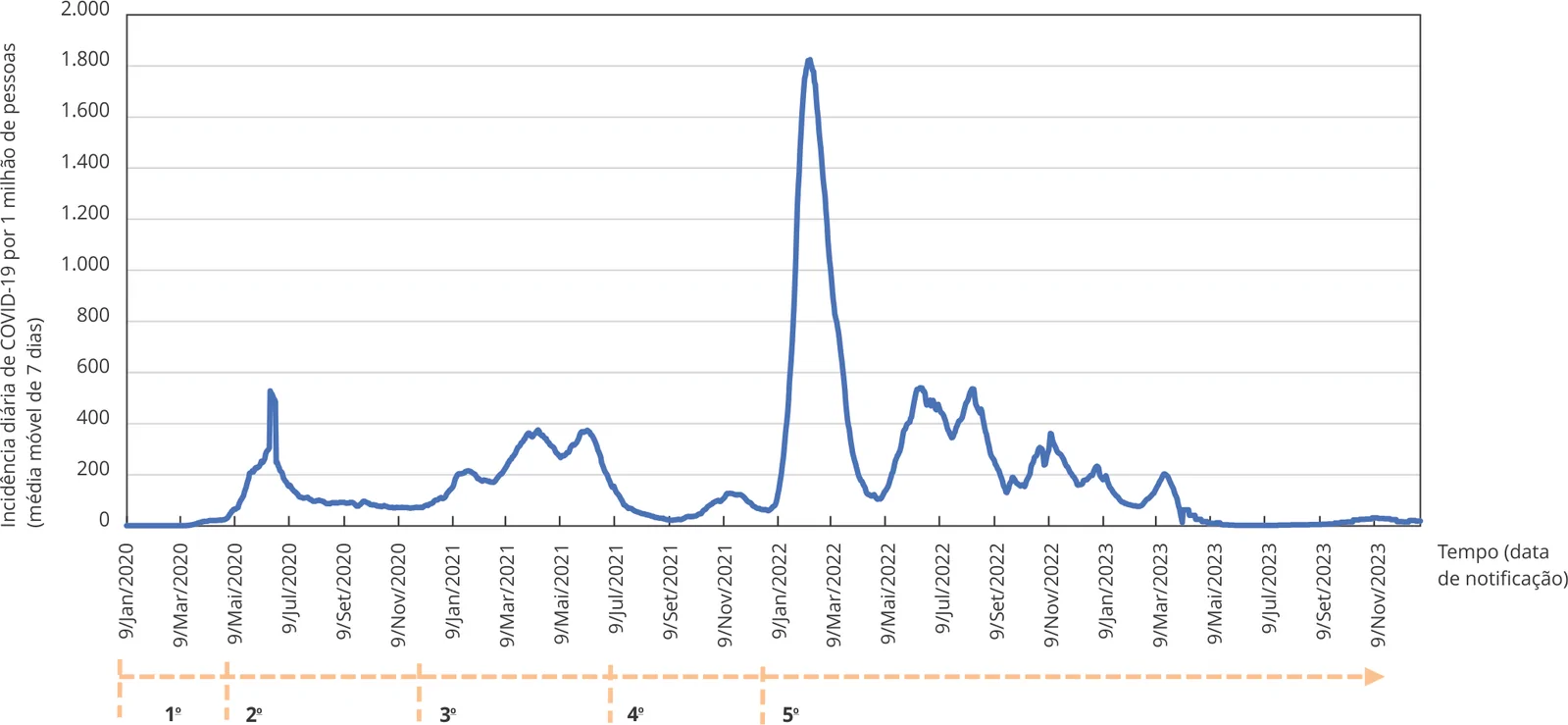
Incidência diária de COVID-19 por 1 milhão de pessoas (média móvel de 7 dias). Chile, 2020-2023.

A trajetória de enfrentamento à COVID-19 no Chile foi analisada em cinco momentos, conforme a situação epidemiológica (média móvel de casos) (Figura 1); a resposta setorial, abrangendo contexto político, atores, principais medidas sanitárias e de apoio socioeconômico (Quadro 3).

Fonte: elaboração própria.

**Quadro 3:** Síntese dos momentos da resposta sanitária. Chile, 2020-2023.

COMPONENTES/MOMENTOS EPIDÊMICOS	MOMENTO INICIAL − JANEIRO A MAIO DE 2020	EXPANSÃO, ESTABILIZAÇÃO E DECLÍNIO DA PRIMEIRA ONDA − MAIO A DEZEMBRO DE 2020	AVANÇO DA VACINAÇÃO, AFROUXAMENTO DE MEDIDAS DE CONTROLE E FORMAÇÃO DE UMA SEGUNDA ONDA, SEGUIDA DE PAULATINO DECRÉSCIMO − JANEIRO A JULHO DE 2021	DESACELERAÇÃO DA EPIDEMIA, AUMENTO DA MOBILIDADE SOCIAL E RECONFIGURAÇÃO POLÍTICO-INSTITUCIONAL EM PERÍODO ELEITORAL − JULHO A DEZEMBRO DE 2021	TRANSIÇÃO DE GOVERNO, MUDANÇAS NA RESPOSTA SANITÁRIA E AGENDA DE REFORMAS NO SISTEMA DE SAÚDE − JANEIRO DE 2022 A AGOSTO DE 2023
Contexto político	Instabilidade do governo de Sebastián Piñera no contexto de *estallido social* (ocorrido a partir de outubro de 2019). Tensões quanto à centralização das decisões, seguidas de criação de instâncias consultivas, em resposta a críticas e a demandas por maior participação	Crise sanitária e política que resultou na troca de ministros da saúde. Momento de realização de plebiscito popular com consequente aprovação da formulação de uma nova constituição	Intensificação de tensões políticas, além de período de eleições para cargos do Executivo, Legislativo e para redatores da Constituição	As eleições presidenciais foram realizadas em dois turnos, marcadas por forte polarização política, culminando na vitória de Gabriel Boric e sua coalizão para liderar o Executivo	Transição e consolidação do governo de Gabriel Boric, em contexto de fragmentação no Legislativo e ausência de maioria governista
Situação epidemiológica	Período inicial de inexistência de casos identificados no país, início da detecção e, posteriormente, fase de transmissão comunitária	Aumento expressivo de casos com formação de primeira onda de casos e óbitos. Após o pico, houve tendência de redução seguida de estabilidade	Formação da segunda onda epidemiológica que gradualmente sofreu tendência de decréscimo	Formação de uma terceira onda epidemiológica, seguida de redução progressiva de casos, internações e mortes por COVID-19	Formação de nova curva de casos e óbitos, em maior parte, atribuídos à circulação da variante Ômicron, com posterior redução
Atores de relevância nas políticas sanitárias	OMS, Ministério da Saúde, Colégio Médico, Associações de Municípios, Universidades e especialistas do meio acadêmico, Conselho Assessor para COVID-19, Mesa Social COVID-19	Atores do momento anterior, acrescidos de: Conselho Assessor Científico Vacina COVID-19, Secretarias Regionais Ministeriais de Saúde (SEREMIs), Direções de Serviços de Saúde, Autoridades gestoras da APS	Atores dos momentos anteriores, com destaque para o Instituto de Saúde Pública do Chile (ISP)	Atores dos momentos anteriores.	Atores dos momentos anteriores, destacando-se: a OPAS/OMS, o Fonasa, autores gestores da APS; acrescidos das ISAPRES, da Comissão Nacional de Resposta Pandêmica COVID-19 e do Conselho Assessor da Atenção Primária Universal
Condução da resposta sanitária nacional	Centralização de decisões na autoridade sanitária e no governo nacional, refletindo-se na execução de ações, seguida da conformação de instâncias consultivas, como o Conselho Assessor e a Mesa Social COVID-19 - em resposta a tensões políticas	Paulatina descentralização de atribuições e de recursos financeiros para níveis subnacionais, com maior impulso a partir da implementação da TTA, destacando a atuação territorial das SEREMI e da APS, bem como esforços para assegurar a vacinação	Continuidade de políticas nacionais anteriores conduzidas sob maior articulação intergovernamental, destacando as amplas iniciativas para a rápida vacinação da população	Valorização da ampla cobertura de vacinação e desafios quanto ao elevado incremento da lista de espera na assistência à saúde, sobretudo, para procedimentos eletivos	Estímulo à dose de reforço de vacinas; propósito de mudança da governança da resposta sanitária
Principais medidas sanitárias	- Delimitação de ações para a Preparação <- Declarações de alinhamento com a OMS e o RSI e anúncio de cooperação com a China para a gestão da crise <- Publicação de Alerta Sanitário pelo Ministério da Saúde (Minsal) <- Primeiras ações de controle em aeroportos e instalação de aduanas sanitárias no país <- Declaração do *Estado de Excepción Constitucional de Catástrofe* <- Restrições de mobilidade populacional <- Fechamento de fronteiras internacionais <- Suspensão de atividades escolares presenciais <- Conformação do *Consejo Asesor* para COVID-19 e da Mesa Social COVID-19 <- Constituição da *Red Integrada de Salud COVID-19*	- Quarentena total de *comunas* do país <- Revisão de critérios de contabilização de óbitos por COVID-19, em alinhamento a recomendações da OMS, considerando casos prováveis houve aumento de registros <- Pressão por mudanças na resposta com tensionamentos para o reforço das ações de vigilância em saúde e maior participação da − APS <- Publicação da *Estrategia Nacional de Testeo, Trazabilidad y Aislamiento* (TTA) <- Iniciativas para garantia de vacinas <- Publicação do *Plan Paso a Paso, nos Cuidamos* <- coordenação nacional da restrição ou flexibilização da mobilidade populacional <- Em dezembro de 2020, começou a vacinação de profissionais de saúde	- Difusão do *Plan Nacional de Vacunación* <- Constituição e divulgação da plataforma *Me* *Vacuno para* registros de vacinação <- Flexibilização de ações para controle de mobilidade populacional <- Criação do *Pase de Mobilidad* <- Prorrogação do *Estado de Excepción Constitucional de Catástrofe*	- Atualização do *Plan Paso a Paso, nos cuidamos* <- Retorno às atividades educacionais <- Implantação da dose de reforço da vacina <- Fim do *Estado de Excepción Constitucional de Catástrofe* <- Destaques aos legados da resposta: vacinação e capacidade diagnóstica	- Atualização dos critérios de classificação e registro de óbitos por COVID-19, em alinhamento à OMS, com aumento de indicadores de mortalidade em contexto de reorganização da resposta sanitária <- Aprovação, aquisição de lotes, distribuição e publicização de doses de reforço da vacina <- Criação da *Comisión Nacional de Respuesta Pandémica* <- Inclusão da vacina Bivalente no PNI <- Publicação do *Plan Seguimos Cuidándonos, Paso a Paso* <- Fim do Alerta Sanitario <- Instituição do Copagamento Zero <- Implantação da APS Universal
Medidas de apoio social e econômico	Educação − Plataforma digital *Aprendo en línea* − aprendizagem remota e cestas básicas para estudantes. Trabalho/Emprego/Renda − *Ley de Protección del Empleo e Seguro de Cesantía* − seguro desemprego *Bono de Emergencia Covid-19* - renda. Ingreso Familiar de Emergencia (IFE) − renda *Plan Alimentos para Chile* − cestas básicas. Empresas − FOGAPE COVID − *Fondo de Garantía para Pequeños y Medianos Empresarios*	Educação − *Aprendo en Casa* − oferta de materiais educativos e atividades a estudantes de escolas rurais e/ou sem conectividade com internet *Aprendo TV* − transmissão de conteúdos educativos Reativação econômica: *Plan Paso a Paso Chile se Recupera* − impulso ao emprego/postos de trabalho, obras de infraestrutura e créditos a pequenas e médias empresas Trabalho/Emprego/Renda − Postnatal de Emergencia e Bono Clase Media − renda *Subsidio al Empleo* − *Subsidio al Regreso y Subsidio a Contratación*	Educação − *Yo confío em mi escuela; Plan Chile Recupera y Aprende, Plan Paso a Paso: Apertura de Establecimientos Educacionales. 2021; Protocolo de Medidas Sanitarias para Establecimientos Educacionales.* Trabalho/Emprego/Renda − *FOGAPE Reactiva; Extensión Postnatal de Emergencia; Ingreso Familiar de Emergencia (IFE) Universal.* Empresas − *Bono PYMEs 2021*	Trabalho/Emprego/Renda − *FOGAPE Reactiva; Ingreso Familiar de Emergencia (IFE) Universal*	Trabalho/Emprego/Renda - *FOGAPE “Chile Apoya”; Aporte Canasta Básica de Alimentos; Bono Chile Apoya Invierno; Bolsillo Familiar Electrónico (BFE); Ingreso Familiar de Emergencia (IFE) Laboral.* Saúde − *Copago Cero*

APS: atenção primária à saúde; Fonasa: Fundo Nacional de Saúde; ISAPRES: Instituições de Saúde Previdenciária; ISP: Instituto de Saúde Pública; OMS: Organização Mundial da Saúde; OPAS: Organização Pan-Americana da Saúde; PNI: Programa Nacional de Imunizações; RSI: Regulamento Sanitário Internacional; SEREMI: Secretarias Regionais Ministeriais de Saúde; TTA: testagem, rastreamento e isolamento.

Fonte: elaboração própria.

O primeiro momento, entre janeiro e maio de 2020, abrangeu a preparação inicial e a introdução de medidas de contenção. Diante do alerta internacional para uma possível emergência em saúde pública ^18^ ^,^ ^19^, o governo declarou-se alinhado à Organização Mundial da Saúde (OMS), decretou Alerta Sanitário e apresentou um plano nacional de preparação. Com a detecção de casos e a posterior situação de transmissão comunitária, declarou-se estado de exceção constitucional de catástrofe, seguido de restrições de mobilidade e fechamento de fronteiras internacionais. 

No quadro político observava-se a fragilidade da confiança social e da representatividade governamental, enquanto diferentes atores assumiram posicionamentos técnicos e políticos ao passo que conflitos em torno do enfrentamento da epidemia repercutiram também na institucionalidade regional/local (E12, E13). Destacam-se reivindicações por participação nas decisões, sobressaindo o Colégio Médico, associações municipais e especialistas do meio científico ^18^ ^,^ ^20^ ^,^ ^21^. Durante as tensões, instituíram-se instâncias consultivas que incorporaram atores críticos às decisões governamentais como o Conselho Assessor para COVID-19 do Minsal e a Mesa Social COVID-19, vinculada ao Ministério do Interior e Segurança Pública (MISP). Ambas tiveram papel relevante na resposta sanitária, inclusive, na produção de informações em saúde, destacando-se a atuação da Sub Mesa de Dados COVID-19, vinculada à última. 

Acerca das medidas sanitárias, destaca-se a criação de residências sanitárias, parcialmente viabilizadas por convênios com o setor privado, ampliadas ao longo do tempo e alvo de contestações quanto à implantação. Ademais, investiu-se na capacidade de vigilância laboratorial, com o desenvolvimento de técnicas de sequenciamento genômico pelo ISP do Chile, com o apoio do Minsal. A comunicação com a população ocorreu principalmente por comunicados à imprensa nacional, além de iniciativas regionais e locais (E4). Já a capacidade hospitalar constituiu um eixo fundamental, ampliada pela compra de equipamentos e insumos, reconversão de leitos e, sobretudo, pela criação da Rede Integrada de Saúde COVID-19, que unificou a gestão de leitos públicos e privados sob a coordenação central da Subsecretaria de Redes Assistenciais do Minsal. 

Entre as medidas mais criticadas estavam as quarentenas estratégicas e dinâmicas, contestadas por cientistas, organizações sociais e meios de comunicação pela proximidade entre territórios com diferentes restrições (E3) e pela insuficiência de políticas de apoio social e econômico (Quadro 3), dificultando a adesão da população (E2, E3). Também foi alvo de críticas o Plano Retorno Seguro, lançado em abril de 2020, sob a justificativa de crise econômica e suposta estabilidade do sistema de saúde. Diante da situação epidemiológica (E9), o governo foi acusado de priorizar interesses corporativos e econômicos em detrimento da saúde. Sobressaia ainda a ausência de amplo sistema de testagem, rastreamento e isolamento, fragilidades na comunicação de risco e reivindicações das associações municipais por maior acesso a informações epidemiológicas para a atuação da APS. A tensão foi intensificada em maio de 2020, com o aumento de casos e o colapso do sistema de saúde de Santiago. 

O segundo momento da resposta sanitária, entre maio e dezembro de 2020, foi marcado pelo crescimento da curva (Figura 1), com o confinamento de localidades, embora análises da Sub Mesa de Dados tenham demonstrado limitações desta medida, com pouca redução da movimentação populacional ^22^. Persistia uma baixa confiança social na autoridade sanitária nacional. No pico de contágios e mortes, cientistas de distintas instituições publicaram carta defendendo mudanças e argumentando que a ênfase na capacidade hospitalar não interrompia as cadeias de transmissão. A intensificação da crise sanitária e política aconteceu diante de situação epidemiológica crítica e de questionamentos quanto à transparência e à consistência dos dados publicizados, culminando na substituição do ministro da saúde Jaime Mañalich por Enrique Paris, em junho de 2020 ^23^. Naquele período, o governo revisou critérios para os registros de mortes por COVID-19, passando a incluir óbitos prováveis e suspeitos. A falta de transparência tornou-se um dos principais eixos de contestação ao governo.

Nesse cenário, a resposta sanitária passou por reconfiguração com a publicação, pelo Minsal, da Estratégia Nacional para Testagem, Rastreamento e Isolamento (TTA, acrônimo em espanhol), que ampliou a participação da APS (E1, E2, E12) e impulsionou a articulação entre SEREMIs, Serviços de Saúde e unidades assistenciais (E11, E12, E13, E14). Sua implantação fortaleceu o financiamento estatal, inclusive, em sistemas de informação em saúde, como o EPIVIGILA ^24^, relevantes para a resposta (E4, E5, E6, E9, E12, E13). Também foi publicado o “*Plan Paso a Paso, Nos Cuidamos*” para a coordenação nacional das restrições de mobilidade. Contudo, pesquisadores e membros da Mesa Social COVID-19 apontaram a persistência de limitações no rastreamento, na confiabilidade dos dados epidemiológicos e a participação ainda incipiente da APS ^25^. 

Paralelamente, avançaram as iniciativas voltadas à vacinação contra a COVID-19, como a criação do Conselho Assessor Científico Vacina COVID-19 e o anúncio do primeiro ensaio clínico no país, em parceria entre a Sinovac Biotech (China), o Minsal, o MCTCI e instituições de ensino privadas. Ao final de 2020, destacavam-se a autorização do uso emergencial do imunizante Pfizer (Estados Unidos)/BioNTech (Alemanha) pelo ISP, a chegada de lotes, a imunização de profissionais de saúde ^26^ e o Plano Nacional de Vacinação, cujo financiamento e distribuição envolveram negociações entre Minsal, MISP e associações de prefeitos. Posteriormente, o ISP aprovou novos imunizantes (E5) e adotou-se o Passe de Mobilidade, com concessões de permissões de mobilidade para estímulo à adesão vacinal (E2). 

O período também foi marcado pela realização de um plebiscito ^27^ que aprovou a formulação de uma nova Constituição, evidenciando a continuidade das tensões em torno das responsabilidades estatais e garantia de direitos sociais. No âmbito sanitário, as fronteiras do país estiveram fechadas entre março e novembro de 2020, com posterior flexibilização diante da melhora do cenário epidemiológico, seguida de novo reforço com a identificação da variante Alfa. 

No terceiro momento, entre janeiro e junho de 2021, os avanços da imunização geraram expectativas de superação da crise sanitária, acompanhadas de abrandamento de regras de mobilidade, associadas a ações de vigilância em saúde, como o reforço da estratégia TTA, com novos centros de rastreamento, ampliação de residências sanitárias e incorporação de testes de antígenos. Nesse contexto, observou-se um aumento de casos nos primeiros meses de 2021, configurando uma segunda onda epidemiológica nacional (Figura 1). Especialistas advertiram para a possível indução governamental de uma percepção equivocada de controle da epidemia ^28^. Diante da alta ocupação de leitos de UTI e da expansão da variante Delta, foram mantidas as restrições de fronteiras e medidas de vigilância. 

No âmbito político-institucional foi realizada a eleição da Comissão Constituinte, marco caracterizado pela paridade de gênero e valorização de povos originários, incluindo a eleição de uma indígena mapuche para a presidência ^29^. O processo também evidenciou o fortalecimento de candidaturas independentes e o relativo enfraquecimento governista. 

No quarto momento, de julho a dezembro de 2021, observou-se o decréscimo de internações e mortes por COVID-19, com o aumento gradual da mobilidade social e posterior encerramento do estado de exceção constitucional. A conjuntura política foi marcada por eleições presidenciais, legislativas e regionais. A disputa presidencial foi ao segundo turno entre Gabriel Boric, da coalizão Apruebo Dignidad, e José António Kast, da Frente Social Cristiana. 

Entre as medidas sanitárias destacou-se a atualização do “*Plan Paso a Paso, Nos Cuidamos*” com maior flexibilização, novos parâmetros e abrangência territorial, e o Plano Fronteiras Protegidas, criticado por ampliar fluxos e riscos de circulação de variantes ^30^ ^,^ ^31^. Naquele momento, a variante Delta seguia como preocupação pela alta transmissibilidade e gravidade, sobretudo entre não-vacinados. Nesse cenário, o Conselho Assessor para COVID-19 recomendou o reforço do controle de fronteiras, da vigilância em saúde e da imunização. Evidências sobre a efetividade da dose de reforço de vacina aceleraram sua implantação e incorporação ao Passe de Mobilidade.

No âmbito científico-tecnológico, um importante avanço foi o acordo com a Sinovac para a instalação de indústria de imunizantes e centro de pesquisas e desenvolvimento, indicando a retomada futura da produção nacional. 

Com a estabilidade epidemiológica e a alta cobertura vacinal, foi encerrado o estado de exceção constitucional de catástrofe, mantendo-se o Alerta Sanitário e reduzindo-se estruturas e contingente mobilizados para a resposta. Posteriormente, surgiu uma terceira onda epidemiológica que, após estabilização, foi seguida pela suspensão do financiamento municipal da estratégia TTA, decisão questionada por possível descontinuidade ^32^. 

A emergência da variante Ômicron na África do Sul levou a mudanças de diretrizes de vigilância e à manutenção de controle nas fronteiras internacionais que foram flexibilizadas, posteriormente, mediante a comprovação de reforço da vacina.

Ao final de 2021, a vacinação consolidava-se como o marco da resposta sanitária chilena, associada à capacidade de negociação de imunizantes, à participação em ensaios clínicos, à aprovação regulatória do ISP, à cultura social favorável às vacinas, à atuação da APS e ao Passe de Mobilidade. Persistiram, contudo, desafios como a demanda reprimida por procedimentos eletivos (E1, E10), inclusive vinculados às GES, recolocando em debate a ampliação da atenção especializada e hospitalar e o enfrentamento de questões estruturais. No âmbito político, a vitória de Gabriel Boric, em dezembro de 2021, instaurou um novo ciclo. 

### Transição governamental, mudanças na resposta sanitária e agenda de reformas no sistema de saúde

O quinto momento, entre janeiro de 2022 e agosto de 2023, correspondeu ao quarto ano da epidemia de COVID-19, no qual a resposta sanitária chilena assumiu paulatina transição, caracterizada pela alta vacinação e flexibilização progressiva das medidas de controle sanitário. A vacinação avançou com a integração de doses de reforço, embora tenha havido, em princípios de 2022, a formação de nova curva epidemiológica, atribuída à circulação da variante Ômicron. Muito embora a COVID-19 permanecesse como questão sanitária relevante, sua gestão passou a ocorrer em um panorama de maior proteção imunológica da população e menor gravidade clínica, com a crescente retomada da vida social. 

Já no início de 2023, a ampla imunização da população refletiu na redução de indicadores de morbidade e mortalidade por COVID-19. O momento estável levou, em maio, à declaração do fim da Emergência de Saúde Pública de Importância Internacional de COVID-19 pela OMS. Em consequência, no Chile foram flexibilizadas diversas exigências sanitárias, incluindo regras para a entrada no país, bem como a contínua redução de casos e mortes veio a culminar no encerramento do Alerta Sanitário em agosto de 2023. 

A quarta curva epidemiológica, formada nos primeiros meses de 2022, atingiu um pico de contágios em fevereiro, com influências sobre o quantitativo de óbitos por COVID-19 ^33^. Os níveis de morbidade foram os mais altos desde o período inicial da epidemia e o governo respondeu com a oferta de uma quarta dose de reforço da vacinação, além da diminuição do tempo de permanência em isolamento e quarentena, considerando que a transmissibilidade se concentrava nos primeiros dias dos sintomas. Já no mês seguinte, o cenário epidemiológico passou ao decréscimo de casos e menor gravidade, além da alta vacinação, configurando condições sanitárias mais favoráveis para uma nova fase de convivência controlada com o vírus. 

Nesse período, o ambiente político foi caracterizado por transição com a posse de Gabriel Boric à presidência. Embora o pico de incidência de casos tenha ocorrido em fevereiro, os elevados registros de óbitos intensificaram os embates políticos. Com a assunção do novo governo, as medidas sanitárias adotadas buscavam reposicionar a resposta.

Destaca-se a mudança de critérios para classificar e registrar as mortes por COVID-19, conforme parâmetros da OMS, o que repercutiu em um significativo aumento dos indicadores de mortalidade. O elevado número de mortes acirrou as disputas em torno da condução da crise, com críticas ao governo Piñera e ao governo Boric sobre transparência, possível subnotificação e uso político dos dados, contribuindo para a crise de confiança na autoridade sanitária.

Também foram anunciadas mudanças na governança da resposta sanitária, pautando-se numa busca por maior participação social e intersetorialidade, bem como na tomada de decisões baseada em evidências. Houve descontinuidade do então Conselho Assessor para a COVID-19, com a criação da Comissão Nacional de Resposta Pandêmica COVID-19, articulada ao Comitê Interministerial de Resposta Pandêmica. E ainda, foi substituído o plano nacional pelo “*Seguimos Cuidándonos Paso a Paso*” que simbolizou uma nova etapa: menos centrada na excepcionalidade da emergência e mais voltada à gestão prolongada dos riscos sanitários, com medidas de proteção individual e coletiva. Ao mesmo tempo, as estratégias abrangeram a incorporação da vacina bivalente ao Programa Nacional de Imunizações e a oferta de autotestes de COVID-19 a baixo custo.

Do ponto de vista institucional, o governo Boric assumiu com uma agenda centrada em direitos sociais e reformas estruturais prioritárias, nas quais a saúde ocupava lugar estratégico. Entre as prioridades destacavam-se o fortalecimento do sistema público de saúde, a criação de um Fundo Universal de Saúde e a universalização da APS. O plano também previa a criação de um Comitê Interministerial de Resposta Pandêmica para analisar os efeitos sociais e econômicos da COVID-19, além de iniciativas voltadas ao fortalecimento da rede de atenção e à redução de listas de espera.

Contudo, a agenda reformista apresentava limites importantes devido à conjuntura política conturbada. Boric assumiu em meio a um Congresso politicamente fragmentado, sem contar com coligações sólidas que o apoiassem. As duas coligações de base − *Apruebo Dignidad* e *Socialismo Democrático* − não tinham votos suficientes para aprovar as reformas estruturais sem estabelecer acordos com os partidos de centro e de direita.

Sendo assim, os processos de reforma do sistema de saúde que se seguiram expressaram tais contradições. Destaca-se o “Copagamento Zero”, publicado em julho de 2022, que eliminou copagamentos para a atenção hospitalar aos usuários dos grupos C e D da Modalidade de Atenção Institucional (MAI) do Fonasa. Sua implantação teve um sentido simbólico e político, ao reforçar o papel do Fonasa como referência para a proteção social. Além da agenda de ampliação da gratuidade no acesso à assistência à saúde. Contudo, foi uma medida de natureza administrativa, interpretada como um avanço incremental que não alcançou a mudança estrutural. 

Outra iniciativa reformista consistiu em processos rumo à universalização da APS, cujo apoio técnico passou a contar com o Conselho Assessor da Atenção Primária Universal, constituído pelo Minsal e composto por diferentes representações como: ex‐ministros da saúde, personalidades políticas, lideranças sociais, acadêmicos, sindicatos, associações profissionais e organismos internacionais. Foi nos primeiros meses de 2023 que a “Reforma APS Universal” foi impulsionada, com o apoio da OMS e da Organização Pan-americana da Saúde (OPAS). Sua implantação começou em sete comunas, unidades político-administrativas locais, contando com o financiamento do Minsal e do Banco Mundial. Considerou-se que esse seria um passo rumo à universalidade da saúde (E15), também abrangendo aperfeiçoamento da obtenção e processamento de informações territoriais (E16). Tais processos foram desenvolvidos com base em sistemas de informação em saúde e georreferenciamento, aspectos amplamente fortalecidos na resposta à COVID-19.

O contexto foi permeado por impasses institucionais e problemáticas regulatórias no setor saúde. Destacou-se o debate sobre as licenças médicas eletrônicas, cujo elevado montante gerou impactos financeiros e questionamentos quanto à legitimidade. Na epidemia, a flexibilização das regras de afastamento laboral buscou apoiar medidas sanitárias, como isolamento e quarentena. Contudo, sua emissão ampliou gastos do Fonasa, principalmente, e das ISAPRES, transformando-se em um problema fiscal, administrativo e regulatório. Com isso, desde 2023 foram reforçadas as medidas para ampliar a sua fiscalização.

Paralelamente, sobressaiu a crise financeira do subsistema privado de seguros de saúde, a crise das ISAPRES, constituída ao longo dos anos, mas agravada em 2022. Essa decorreu, sobretudo, de mecanismos de financiamento baseados na estratificação de riscos, com aumentos tarifários posteriormente considerados abusivos e objetos de judicialização. Como desdobramento, a Suprema Corte estabeleceu uma tabela única de custos e a obrigatoriedade de reembolso dos valores cobrados, intensificando a crise financeira e o risco de insolvência dessas instituições. Isso colocou o governo sob pressão para evitar impactos do setor privado sobre a rede pública, ao mesmo tempo em que avançava na agenda da cobertura universal.

Prosseguindo os processos políticos constituintes desencadeados como resposta à mobilização social de 2019, foi realizado um plebiscito, em setembro de 2022, para a votação de uma primeira proposta de nova Constituição, rejeitada por cerca de 62% dos votos. Ressalta-se que essa primeira proposta continha transformações estruturais, como o reconhecimento do Estado plurinacional, mudanças nos sistemas político e judicial do Chile e a instituição de um sistema nacional de saúde universal, público e integrado, com a APS como a porta de entrada preferencial. Tal rejeição reuniu setores partidários de direita e parcelas da centro-esquerda. Na sequência, um novo processo constituinte foi conduzido, sendo a sua proposta submetida a plebiscito em dezembro de 2023. Em sentido oposto ao anterior, esse reservava ao Estado um papel subsidiário e enfatizava a coparticipação público-privada no setor saúde, prevendo a instituição de um Plano de Saúde Universal. Sua rejeição expressou, assim, as dificuldades de construção de consenso político e social em torno de um novo pacto constitucional para o país. 

### Discussão: da resposta à COVID-19 às Reformas do Sistema de Saúde

Os primeiros anos da resposta sanitária à COVID-19 no Chile evidenciaram um importante paradoxo entre capacidade institucional e instabilidade político-social. Embora a resposta tenha sido reconhecida internacionalmente pela gestão centralizada de leitos, ampliação diagnóstica e acelerada vacinação, desenvolveu-se sob baixa legitimidade governamental, centralização decisória ^34^ e limitada participação social, com morosidade de apoio socioeconômico ^35^ e, principalmente, persistência dos conflitos associados ao *estallido social* de 2019 ^34^.

A epidemia de COVID-19 impôs uma “trégua” temporária à revolta social iniciada em 2019, reconfigurando a crise política chilena sem interromper suas tensões estruturais. A baixa legitimidade do governo Piñera e as contradições da resposta sanitária impactaram a sua aprovação e evidenciaram limites do Estado chileno na proteção social e garantia de direitos, fortalecendo debates em torno de um Estado de bem-estar social universal e do ciclo político associado à eleição de Gabriel Boric. 

Destacaram-se capacidades estatais preexistentes fundamentais à resposta sanitária, como o Programa Nacional de Imunizações, a capacidade de coordenação do Ministério da Saúde e a rede de laboratórios clínicos, fortalecida nacionalmente. A estratégia vacinal constituiu o principal sucesso técnico e político da resposta, apoiada por negociações internacionais, diversificação de contratos, articulação diplomática e realização de ensaios clínicos. Ainda assim, houve críticas quanto à transparência contratual, além de debates acerca da efetividade dos imunizantes.

As disputas em torno da produção e transparência de dados em saúde demonstraram que os sistemas de informação constituíram a dimensão central da governança sanitária. A resposta fortaleceu ações territoriais conduzidas por SEREMIs e APS, incluindo rastreamento, monitoramento domiciliar, testagem e vacinação (E1, E2, E7, E8) e alinhadas às recomendações internacionais ^36^ ^,^ ^37^. Contudo, a integração da APS foi considerada tardia ^34^ ^,^ ^35^.

A criação da “Rede Integrada de Saúde COVID-19”, ao centralizar a gestão de leitos públicos e privados sob a coordenação estatal, representou uma redução temporária da segmentação do sistema de saúde. O protagonismo do setor público durante a epidemia ampliou a legitimidade de reformas posteriores voltadas ao fortalecimento da universalidade, como o “Copagamento Zero” e a “APS Universal”. 

Em contraste, os processos constituintes de 2022 e 2023 fracassaram em estabelecer um novo pacto social para a saúde, incluindo a rejeição da proposta de um Sistema Nacional de Saúde Universal. Diante desse cenário, reformas inicialmente associadas a mudanças estruturais passaram a assumir caráter incremental e tecnicamente orientado, implementadas por mecanismos administrativos e programáticos, em um movimento denominado “realismo pragmático”.

Apesar dos avanços, persistiram os desafios relacionados à capacidade assistencial e ao elevado gasto direto das famílias com saúde. Em 2023, o desembolso direto alcançou 35% do gasto em saúde no Chile, entre os maiores índices da Organização para a Cooperação e Desenvolvimento Econômico (OCDE) ^38^. Paralelamente, a “APS Universal” passou a ser implementada por projetos-piloto com financiamento misto, refletindo adaptações às restrições políticas do pós-epidemia. A crise das ISAPRES, agravada em 2022, acelerou a migração de usuários ao Fonasa e reforçou o protagonismo do setor público.

Os legados da epidemia, associados ao fortalecimento da vigilância em saúde e à maior legitimidade do setor público, permaneceram centrais. Contudo, seguem as questões sobre a capacidade das reformas incrementais reduzirem as iniquidades, ampliarem o acesso e superarem a segmentação do sistema. 

Em 2026, começou o governo de José Antonio Kast (2026-2030), com a agenda voltada à ampliação da participação privada no atendimento das listas de espera. Em um sistema historicamente segmentado, com limitada oferta pública e elevado gasto direto das famílias, essa orientação pode aprofundar as desigualdades. Em contraste com a agenda de fortalecimento da APS e da universalidade do governo Gabriel Boric, o novo governo recoloca o debate da saúde sob um enfoque mercadológico, abrindo questões sobre os impactos dessas mudanças na capacidade estatal e na sustentabilidade de reformas orientadas à universalidade. 

Como limitações do estudo, destacamos o perfil dos entrevistados, concentrado em atores institucionais da gestão da saúde, sem incluir profissionais da assistência ou usuários, e o recorte temporal centrado nos primeiros anos da resposta sanitária. Sugere-se um aprofundamento de análises futuras sobre os processos de reformas em curso.

## Data Availability

As fontes de informação utilizadas no estudo estão indicadas no corpo do artigo.
